# Consumption of meat in relation to physical functioning in the Seniors-ENRICA cohort

**DOI:** 10.1186/s12916-018-1036-4

**Published:** 2018-04-05

**Authors:** Ellen A. Struijk, José R. Banegas, Fernando Rodríguez-Artalejo, Esther Lopez-Garcia

**Affiliations:** 1 Department of Preventive Medicine and Public Health, School of Medicine, Universidad Autónoma de Madrid, Avda. Arzobispo Morcillo, 4, 28029 Madrid, Spain; 2 CIBERESP (CIBER of Epidemiology and Public Health), Madrid, Spain; 3 IMDEA Food Institute, CEI UAM+CSIC, Madrid, Spain

**Keywords:** Physical functioning, Meat intake, Red meat, Processed meat, Poultry, Elderly, Agility, Mobility, SPPB

## Abstract

**Background:**

Meat is an important source of high-quality protein and vitamin B but also has a relatively high content of saturated and *trans* fatty acids. Although protein and vitamin B intake seems to protect people from functional limitations, little is known about the effect of habitual meat consumption on physical function. The objective of this study was to examine the prospective association between the intake of meat (processed meat, red meat, and poultry) and physical function impairment in older adults.

**Methods:**

Data were collected for 2982 participants in the Seniors-ENRICA cohort, who were aged ≥60 years and free of physical function impairment. In 2008–2010, their habitual diet was assessed through a validated computer-assisted face-to-face diet history. Study participants were followed up through 2015 to assess self-reported incident impairment in agility, mobility, and performance-based lower-extremity function.

**Results:**

Over a median follow-up of 5.2 years, we identified 625 participants with impaired agility, 455 with impaired mobility, and 446 with impaired lower-extremity function. After adjustment for potential confounders, processed meat intake was associated with a higher risk of impaired agility (hazard ratio [HR] for highest vs. lowest tertile: 1.33; 95% confidence interval [CI]: 1.08–1.64; *p* trend = 0.01) and of impaired lower-extremity function (HR for highest vs. lowest tertile: 1.31; 95% CI: 1.02–1.68; *p* trend = 0.04). No significant associations were found for red meat and poultry. Replacing one serving per day of processed meat with one serving per day of red meat, poultry, or with other important protein sources (fish, legumes, dairy, and nuts) was associated with lower risk of impaired agility and lower-extremity function.

**Conclusions:**

A higher consumption of processed meat was associated with a higher risk of impairment in agility and lower-extremity function. Replacing processed meat by other protein sources may slow the decline in physical functioning in older adults.

**Electronic supplementary material:**

The online version of this article (10.1186/s12916-018-1036-4) contains supplementary material, which is available to authorized users.

## Background

Due to the increase in life expectancy over recent decades, older adults are becoming the largest segment of the population [1]. Consequently, more people are suffering from limitations in physical and cognitive functioning as well as disability, which have a major impact on the quality of life and use of health services [2]. To ensure that people not only live longer but also have healthier lives, more knowledge is needed about the determinants of physical functioning. Some of the factors that have previously been associated with functioning impairment include chronic diseases, inflammation, oxidative stress, waist circumference, and modifiable health behaviors, including several dietary components [3–7].

Meat is an important source of high-quality protein because it contains large quantities of essential amino acids [8]. Therefore, higher animal protein intake has been associated with better physical functioning and with other age-related conditions, such as muscle strength and frailty among older populations [9–14]. In addition to protein, meat contains other nutrients, such as B vitamins, which may also be beneficial to physical functioning [15]. On the other hand, meat has a relatively high content of saturated and *trans* fatty acids [8]. The latest statement from the American Heart Association on dietary fats and cardiovascular disease concluded that lowering the intake of saturated and *trans* fats and replacing them with unsaturated fats reduces the incidence of cardiovascular disease [16]. In addition, a recent meta-analysis showed that red meat and processed meat consumption was associated with increased total, cardiovascular, and cancer mortality [17], with less conclusive results for unprocessed meat among studies conducted outside of the U.S. Therefore, understanding the health effect of habitual meat consumption in older adults is of great interest given the high prevalence of both cardiometabolic risk factors and malnutrition in this population, which produces loss of muscle mass and subsequent frailty and disability [18–21]. To our knowledge, only the Framingham Offspring study has assessed the association between red and white meat consumption and physical function; participants with a higher intake of red meat or poultry and fish had a lower risk of developing two or more functional impairments during the follow-up, although the association was only significant in those with high levels of physical activity [22].

The aim of this study was to investigate prospectively meat consumption (processed meat, red meat, and poultry) in association with self-reported (agility and mobility) and performance-based (lower-extremity function) domains of physical functioning in a European cohort of community-dwelling older adults.

## Methods

### Study design and participants

Data were taken from the Seniors-ENRICA cohort, whose methods have been reported elsewhere [23, 24]. In brief, the cohort was derived from the ENRICA study, which ran from 2008 to 2010 among individuals representative of the non-institutionalized adult population of Spain. The study participants, who were aged 60 years or older, comprised the Seniors-ENRICA cohort (*n* = 3289). At baseline, information on socio-demographic variables, lifestyle, health status, and morbidity was collected through a phone interview. Also, details of their food consumption were obtained, and a physical examination was performed by trained staff in their homes. Two waves of data collection were performed to update the information about the cohort, the first in 2012 and the second in 2015. In total, 1291 participants were lost during the follow-up (675 in the first wave and 616 in the second) and 177 deaths were identified (95 in the first wave and 82 in the second). The participants lost to follow-up were mostly women, had a lower educational level, and were more often obese. Their intake of the different categories of meat was similar in both groups.

Before our analysis, we excluded participants for whom we had no information on diet at baseline or during the follow-up or with an implausibly high or low energy intake (outside the range of 800–5000 kcal/day for men and 500–4000 kcal/day for women), leaving 2982 participants. Furthermore, we excluded those with impaired physical functioning at baseline or those for whom we had no information on physical function at baseline or during follow-up, depending on the domain investigated (the definitions of the three physical function domains are described below). Therefore, the number of participants available for the analysis varied for each domain of physical functioning investigated: 2681 for agility, 2732 for mobility, and 2982 for lower-extremity function, as measured using the Short Physical Performance Battery (SPPB). Since we did not perform the SPPB at baseline, we excluded participants who were frail at baseline as a proxy of impaired lower-extremity function. Frailty at baseline was defined with the Frail scale [25]. Parts of the methods have been described previously [15]. Study participants gave written informed consent. The study was approved by the Clinical Research Ethics Committee of La Paz University Hospital in Madrid.

### Study variables

#### Diet

Information on food consumption was obtained through a validated computer-assisted face-to-face diet history, which was developed from the one used in the EPIC cohort study in Spain [26]. This instrument records the habitual consumption of 880 foods in the preceding year, and includes a set of photographs to help in the quantification of food portions. Energy intake and the intake of other nutrients were estimated using standard food composition tables for Spain [26]. Food consumption was assessed at baseline and again in 2012, and the cumulative average was used for this study. The validity of the diet history was assessed against seven 24-hour recalls over 1 year of 132 men and women, and showed good correlation coefficients for meat intake (*r* = 0.66) [26]. We grouped the different types of meat recorded into three mutually exclusive categories: processed meat (including bacon, salami, and sausages), red meat (including beef, lamb, and pork), and poultry (including several types of fowl and rabbit). Of note, in Spain, poultry accounts for most white meat consumption. Organ meat has not been taken into account due to its low intake.

#### Physical function

We assessed three different domains of physical functioning: agility, mobility, and lower-extremity function. Participants were deemed to have impaired agility when they answered “a lot” to the following question from the Rosow and Breslau scale [27]: “On an average day with your current health, would you be limited in bending and kneeling?” The categories of response were “yes, a lot,” “yes, a little,” and “not at all.” In the same way, impairment in mobility was defined as answering “a lot” to any of the following questions from the Rosow and Breslau scale: “On an average day with your current health, would you be limited in the following activities: (1) picking up or carrying a shopping bag?; (2) climbing one flight of stairs?; (3) walking several city blocks (a few hundred meters)?” Limitation in the lower-extremity function was assessed with the SPPB, which includes three measurements: gait speed across 2.44 m, the ability to rise from a chair five times consecutively, and standing balance using three hierarchical tandem tests [28]. Each component was scored on a four-point scale, and the total score was the sum of the three components (range 0–12). A higher score indicates better physical performance. Although the standard score for functional limitation is ≤9, we used a ≤6-point cut-off to improve the sensitivity.

#### Other variables

At baseline and at the follow-ups in 2012 and 2015, we obtained information on socio-demographic variables, lifestyle, anthropometrics, and disease history. Educational level was classified into primary, secondary, or university level, and smoking status as never smoked, former smoker, or current smoker. Weight and height were measured under standardized conditions. Body mass index (BMI) was calculated as weight (kg) divided by height squared (m^2^), and classified as <25, 25–29.9, or ≥30 kg/m^2^. Physical activity during leisure time (metabolic equivalent hours/week) was ascertained with the EPIC cohort questionnaire, validated in Spain [29]. Sedentary behavior was approximated by the time (hours/week) spent watching television. Cognitive function was assessed with the Mini-Mental State Examination (MMSE), and cognitive impairment was defined as a MMSE score <23 [30]. Participants also reported the following physician-diagnosed diseases: osteomuscular disease, cardiovascular disease, cancer, chronic lung disease, and depression requiring treatment.

All-cause deaths were ascertained by a computerized search of the National Death Index, which contains information on the vital status of all residents in Spain [31]. This information was available for 99.9% of the cohort.

### Statistical analysis

A Cox proportional hazard analysis was performed to determine the hazard ratios (HRs) and the 95% confidence intervals (CIs) for the relationship between habitual meat consumption and incident impairment in physical functioning. The study sample was categorized into tertiles of meat intake, using the first (lowest intake) as the reference category. Additionally, the analyses were repeated for a (continuous) increase of 100 g/day in meat intake to assess a linear dose–response relation. We conducted analyses for each different type of meat and for the different domains of physical functioning (agility, mobility, and lower-extremity function).

The duration of follow-up was determined by the period from the date of study entry to the date of assessment of physical limitation, loss to follow-up, death, or the end of the study, whichever came first. Three Cox models were built: the first adjusting for age and sex; a second model with additional adjustment for educational level, smoking status, alcohol intake, energy intake, BMI, sedentary behavior, and morbidity (cognitive decline, osteomuscular disease, cardiovascular disease, cancer, chronic lung disease and depression requiring treatment), to understand their impact on the studied association; and a third model, additionally adjusted for the following food groups: vegetables, legumes, fruit, nuts, cereals, dairy, and fish (all in quintiles of g/day). The variables in the model that had been measured at baseline and in 2012 were updated in the follow-up.

We conducted several sensitivity analyses. A possible modifying effect of sex was tested by using likelihood-ratio tests, which compared models with and without cross-product interaction terms. Given that we found no sex interactions, our results are presented for the total sample. Also, those associations that were found to be significant were stratified by sex, age, and obesity to assess the robustness of the results. Furthermore, because of possible synergistic effects of physical activity and protein intake [22], we additionally stratified by physical activity (above and below the median) and total protein intake (above and below the median). Lastly, we estimated the effect of replacing a serving per day of processed meat by red meat, poultry, or an alternative protein source, by including both forms of consumption as continuous variables in the model. The differences in the beta coefficients were used to estimate the HR and 95% CI of the substitution associations.

The proportionality assumption was checked visually by log minus log plots with no deviations detected. Differences in population characteristics between categories of consumption were assessed by ANOVA followed by Tukey tests for continuous variables, and by the chi-square test for categorical variables. Statistical significance was set at two-tailed *p* < 0.05. Analyses were conducted using SAS software, version 9.4 (SAS Institute Inc.).

## Results

In this population, mean (standard deviation) consumption was 35.0 (34.0) g/day for processed meat, 31.9 (28.2) g/day for red meat, and 33.8 (28.3) g/day for poultry. The correlations between the intake of the different meat categories (in g/day) were: *r* = 0.05, *p* = 0.01 (between processed meat and red meat), *r* = − 0.02, *p* = 0.21 (between processed meat and poultry), and *r* = 0.11, *p* < 0.01 (between red meat and poultry). Participants in the highest tertile of processed meat consumption were significantly younger, were more often men, were more often current smokers, were more physically active, spent more time watching television, had higher energy and alcohol intake, and ate less fruit and fish compared to those in the lowest tertile of consumption. In addition, those in the highest tertile of red meat consumption were more educated and less likely to have cognitive impairment, osteomuscular disease, or depression, and had a higher vegetable consumption than those in the lowest tertile (Table 1).

**Table 1:** Population characteristics of the study participants across tertiles of meat consumption (*N* = 2982)

	Processed meat			Red meat			Poultry		
	Tertile 1	Tertile 2	Tertile 3	Tertile 1	Tertile 2	Tertile 3	Tertile 1	Tertile 2	Tertile 3
Range (g/day)	0.0–18.0	18.1–39.0	39.0–448	0.0–16.4	16.5–36.4	36.4–312.3	0.0–19.0	19.0–39.6	39.7–311
Number of participants	994	994	994	994	994	994	994	994	994
Age (years)	70.2 ± 7.0	68.8 ± 6.4	68.2 ± 6.2*	70.2 ± 7.1	68.9 ± 6.4	68.0 ± 5.9*	69.5 ± 6.8	68.9 ± 6.4	68.7 ± 6.5*
Sex, men (%)	38.0	45.5	56.0*	35.4	44.2	59.9*	42.6	46.8	50.2*
Educational level (%)									
≤Primary	59.8	56.5	55.1	64.6	57.4	49.5*	56.5	58.2	56.7
Secondary	21.7	24.9	24.3	18.7	26.2	26.0	22.4	25.3	23.1
University	18.5	18.6	20.6	16.8	16.5	24.6	21.0	16.5	20.2
Smoking status (%)									
Current smoker	8.8	12.2	15.2*	9.0	12.3	14.8*	12.2	11.5	12.5
Former smoker	26.5	29.0	35.0	28.1	27.5	34.9	26.8	31.2	32.5
Never smoked	64.8	58.9	49.8	63.0	60.2	50.3	61.1	57.4	55.0
Leisure time physical activity (MET hours/week)	21.1 ± 14.2	22.1 ± 15.1	22.5 ± 15.1*	21.0 ± 14.4	22.3 ± 14.6	22.5 ± 15.3*	21.3 ± 14.7	22.0 ± 14.5	22.5 ± 15.3
Watching television (hours/week)	18.7 ± 11.0	19.7 ± 10.7	19.8 ± 11.9*	19.6 ± 11.8	19.5 ± 11.2	19.0 ± 10.7	19.4 ± 11.8	19.6 ± 11.0	19.2 ± 10.9
Energy intake (kcal/day)	1827 ± 470	2007 ± 490	2174 ± 560*	1834 ± 493	1993 ± 487	2180 ± 543*	1941 ± 542	2000 ± 488	2067 ± 543*
Alcohol intake (g/day)	6.6 ± 12.0	9.5 ± 15.5	11.6 ± 17.3*	7.0 ± 13.8	8.7 ± 14.0	11.9 ± 17.2*	9.2 ± 15.6	9.6 ± 15.2	8.9 ± 14.8
BMI (%)									
<25 kg/m^2^	22.1	22.3	17.2*	20.8	22.0	18.9	20.6	22.4	18.6
25–29.9 kg/m^2^	48.3	46.7	45.6	45.6	48.0	46.9	48.6	44.9	47.0
≥30 kg/m^2^	29.6	31.0	37.2	33.6	30.0	34.2	30.8	32.7	34.3
Diagnosed morbidity (%)									
Cognitive impairment^a^	3.4	3.4	2.3	4.9	2.7	1.6*	3.2	3.1	2.7
Osteomuscular disease^b^	52.8	50.1	48.4	53.9	52.9	44.4*	52.0	49.4	49.8
Cardiovascular disease^c^	4.9	5.7	5.6	5.8	4.6	5.9	4.6	6.2	5.4
Cancer	2.5	3.2	3.1	3.0	2.8	3.0	2.1	3.7	3.0
Chronic lung disease	9.4	7.8	8.1	9.4	8.6	7.1	8.6	8.3	8.3
Depression	8.5	8.5	8.7	10.5	7.9	7.1*	9.5	8.6	7.5*
Food consumption (g/day)									
Vegetables	213 ± 141	224 ± 135	219 ± 140	208 ± 136	222 ± 135	227 ± 143*	197 ± 124	218 ± 129	242 ± 156*
Legumes	54.3 ± 63.6	53.6 ± 63.4	55.7 ± 63.6	56.1 ± 68.4	53.5 ± 63.8	53.9 ± 58.0	54.2 ± 64.7	53.1 ± 61.9	56.2 ± 64.1
Fruit	354 ± 200	336 ± 198	321 ± 187*	335 ± 192	344 ± 195	332 ± 199	337 ± 207	324 ± 178	351 ± 199
Nuts	8.9 ± 15.7	9.6 ± 15.5	9.6 ± 16.2	9.9 ± 17.6	9.3 ± 14.4	8.8 ± 15.3	9.3 ± 16.2	10.2 ± 16.1	8.6 ± 15.1
Cereals	207 ± 90	224 ± 88	241 ± 98*	207 ± 89	225 ± 87	240 ± 100*	220 ± 95	222 ± 86	230 ± 98*
Dairy	333 ± 179	331 ± 197	319 ± 187	331 ± 181	327 ± 184	324 ± 198	320 ± 177	332 ± 196	330 ± 191
Fish	70.7 ± 50.5	70.4 ± 43.9	64.7 ± 43.3*	60.1 ± 45.4	68.5 ± 42.7	77.1 ± 48.4*	61.4 ± 43.3	67.3 ± 44.5	77.1 ± 48.8*

For continuous variables, mean and standard deviation are reported

*Statistically different across tertiles (*p* < 0.05), from ANOVA followed by Tukey tests for continuous variables and from chi-square tests for categorical variables

*BMI* body mass index, *MET* metabolic equivalent, *MMSE* Mini-Mental State Examination

^a^Cognitive impairment is defined as a MMSE score < 23

^b^Osteoarthritis, arthritis, and hip fracture

^c^Ischemic heart disease, stroke, and heart failure

During a median of 5.2 years of follow-up, we identified 625 participants with incident impaired agility, 455 with impaired mobility, and 446 with impaired lower-extremity function. Those in the highest tertile of processed meat consumption showed a higher risk of impaired agility (model 3, HR for highest vs. lowest tertile: 1.33 95% CI: 1.08–1.64; *p* trend = 0.01). A 100 g/day increase in processed meat consumption was associated with a 23% higher risk of impaired agility (HR 1.23; 95% CI: 1.00–1.53) (Table 2). Red meat and poultry consumption were not associated with agility limitation.

**Table 2:** HR and 95% CI for the association between meat consumption and impairment in agility

	Meat intake				Continuous
	Tertile 1	Tertile 2	Tertile 3	*P* trend	per 100 g/day
*N* participants	893	894	894		2681
Processed meat					
Mean intake (g/day)	8.5 ± 27.9	27.9 ± 6.1	69.6 ± 38.0		35.3 ± 34.0
Impairment in agility (*n*/person-years)	188/4220	214/4469	223/4360		625/13,049
Model 1	Reference	1.09 (0.90–1.33)	1.38 (1.13–1.68)	0.001	1.35 (1.11–1.64)
Model 2	Reference	1.08 (0.89–1.32)	1.31 (1.07–1.60)	0.01	1.30 (1.05–1.62)
Model 3	Reference	1.14 (0.93–1.39)	1.33 (1.08–1.64)	0.01	1.23 (1.00–1.53)
Red meat					
Mean intake (g/day)	7.5 ± 5.4	26.4 ± 5.9	63.7 ± 27.0		32.5 ± 28.5
Impairment in agility (*n*/person-years)	230/4313	220/4391	175/4345		625/13,049
Model 1	Reference	1.04 (0.87–1.26)	1.03 (0.84–1.26)	0.77	1.02 (0.95–1.08)
Model 2	Reference	1.13 (0.93–1.36)	1.13 (0.92–1.40)	0.22	1.23 (0.88–1.70)
Model 3	Reference	1.15 (0.95–1.40)	1.14 (0.92–1.41)	0.20	1.07 (0.96–1.19)
Poultry					
Mean intake (g/day)	9.1 ± 6.6	28.7 ± 5.5	64.2 ± 28.6		34.0 ± 28.6
Impairment in agility (*n*/person-years)	200/4278	227/4435	198/4336		625/13,049
Model 1	Reference	1.15 (0.95–1.39)	1.04 (0.86–1.27)	0.66	0.93 (0.69–1.25)
Model 2	Reference	1.10 (0.91–1.33)	1.02 (0.84–1.25)	0.83	0.90 (0.66–1.23)
Model 3	Reference	1.14 (0.94–1.38)	1.07 (0.88–1.32)	0.48	0.94 (0.69–1.29)

Model 1 is adjusted for age and sex

Model 2 is adjusted for age, sex, educational level (≤primary, secondary, or university), smoking status (never smoked, former smoker, or current smoker), alcohol intake (quintiles of g/day), energy intake (quintiles of kcal/day), BMI (<25, 25 < 30, ≥30 kg/m^2^), sedentary behavior (quintiles of hours/week watching television), and morbidity (cognitive impairment, osteomuscular disease, cardiovascular disease, cancer, chronic lung disease, or depression)

Model 3 is, additionally, adjusted for vegetables, legumes, fruits, nuts, cereals, dairy, and fish consumption (quintiles of g/day)

*BMI* body mass index, *CI* confidence interval, *HR* hazard ratio

Higher consumption of processed meat was also associated with impaired lower-extremity function (model 3, HR for highest vs. lowest tertile: 1.31; 95% CI: 1.02–1.68; *p* trend = 0.04) (Table 3). Again, neither red meat nor poultry were associated with physical function, as assessed with the SPPB. In addition, none of the meat types was associated with limitations in mobility (Additional file 1: Table S1).

**Table 3:** HR and 95% CI for the association between meat consumption and impaired lower-extremity function

	Meat intake				Continuous
	Tertile 1	Tertile 2	Tertile 3	*P* trend	per 100 g/day
*N* participants	994	994	994		2982
Processed meat					
Mean intake (g/day)	8.2 ± 5.8	27.6 ± 6.1	69.3 ± 38.1		35.0 ± 34.0
Impairment of lower-extremity function, n/person-years	131/4450	153/4778	162/4685		446/13,914
Model 1	Reference	1.14 (0.90–1.44)	1.50 (1.18–1.89)	0.001	1.27 (0.97–1.67)
Model 2	Reference	1.15 (0.91–1.46)	1.37 (1.07–1.75)	0.01	1.12 (0.83–1.52)
Model 3	Reference	1.22 (0.96–1.56)	1.31 (1.02–1.68)	0.04	1.00 (0.72–1.38)
Red meat					
Mean intake (g/day)	7.1 ± 5.3	25.7 ± 5.9	62.8 ± 26.8		31.9 ± 28.2
Impairment of lower-extremity function (*n*/person-years)	166/4504	169/4737	111/4672		446/13,914
Model 1	Reference	1.13 (0.91–1.40)	0.89 (0.69–1.14)	0.43	0.75 (0.49–1.14)
Model 2	Reference	1.19 (0.96–1.49)	0.87 (0.68–1.13)	0.42	0.69 (0.45–1.06)
Model 3	Reference	1.25 (1.00–1.56)	0.86 (0.67–1.12)	0.43	0.70 (0.45–1.06)
Poultry					
Mean intake (g/day)	9.0 ± 6.6	28.6 ± 5.6	64.0 ± 27.8		33.8 ± 28.3
Impairment of lower-extremity function, n/person-years	139/4542	171/4590	136/4590		446/13,914
Model 1	Reference	1.27 (1.02–1.60)	1.03 (0.81–1.30)	0.81	1.24 (0.86–1.80)
Model 2	Reference	1.30 (1.03–1.63)	1.00 (0.79–1.28)	0.99	1.15 (0.79–1.68)
Model 3	Reference	1.34 (1.06–1.69)	1.08 (0.85–1.38)	0.50	1.31 (0.90–1.91)

Model 1 is adjusted for age and sex

Model 2 is adjusted for age, sex, educational level (≤primary, secondary, or university), smoking status (never smoked, former smoker, or current smoker), alcohol intake (quintiles of g/day), energy intake (quintiles of kcal/day), BMI (<25, 25 < 30, ≥30 kg/m^2^), sedentary behavior (quintiles of hours/week watching television), and morbidity (cognitive impairment, osteomuscular disease, cardiovascular disease, cancer, chronic lung disease, and depression)

Model 3 is, additionally, adjusted for vegetables, legumes, fruits, nuts, cereals, dairy, and fish consumption (quintiles of g/day)

*BMI* body mass index, *CI* confidence interval, *HR* hazard ratio

**Fig. 1:**
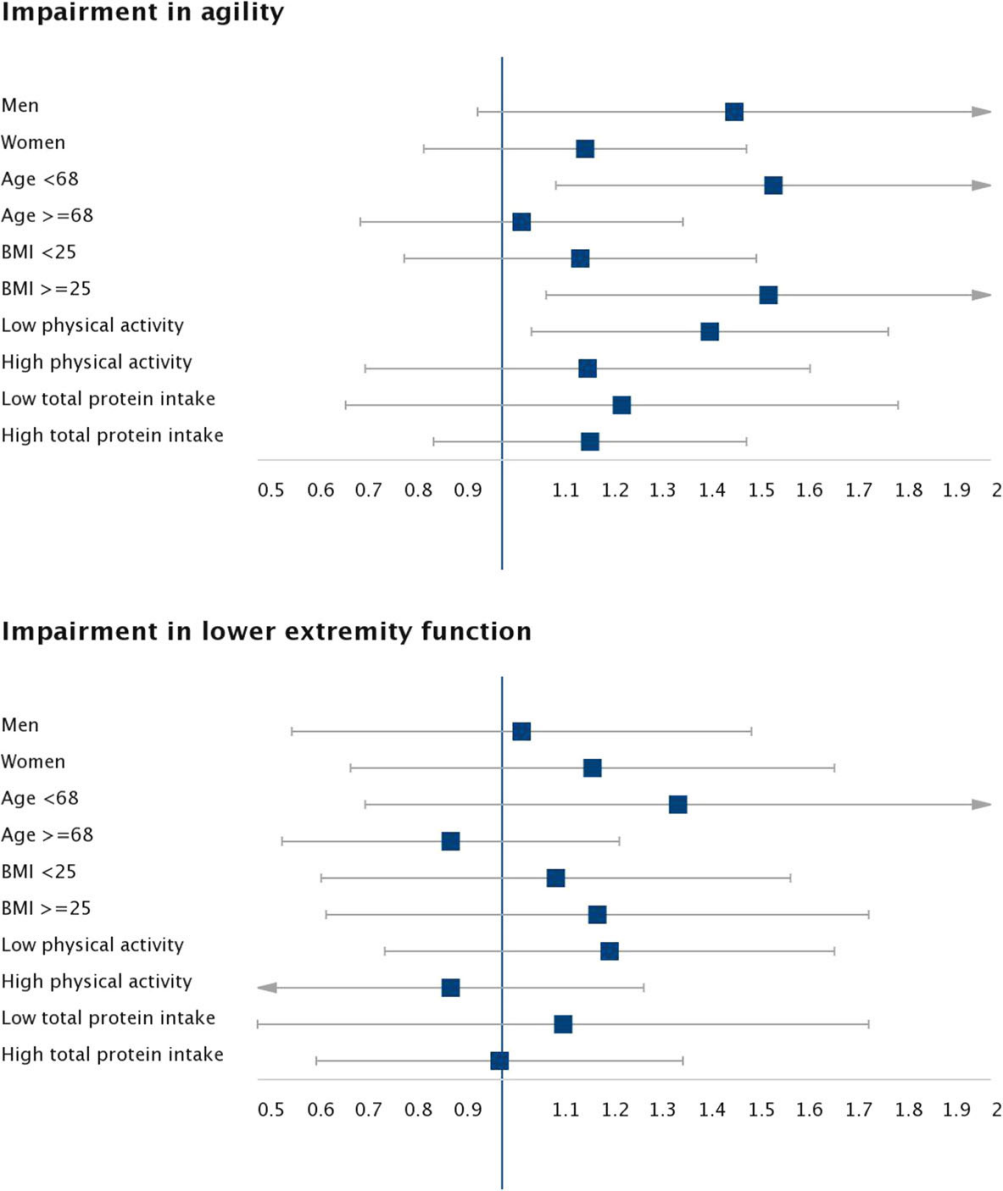
Stratified analyses for the associations between processed meat consumption and impaired physical function during a median follow-up of 5.2 years. Models were adjusted for age, sex, educational level (≤primary, secondary, or university), smoking status (never smoked, former smoker, or current smoker), alcohol intake (quintiles of g/day), energy intake (quintiles of kcal/day), BMI (<25, 25 < 30, ≥30 kg/m^2^), sedentary behavior (quintiles of hours/week watching television), morbidity (cognitive impairment, osteomuscular disease, cardiovascular disease, cancer, chronic lung disease, or depression), and for vegetables, legumes, fruits, nuts, cereals, dairy, and fish consumption (quintiles of g/day). BMI body mass index

Figure 1 shows the associations of processed meat consumption with impaired agility and lower-extremity function stratified by socio-demographic variables and health behaviors. We found no significant differences between the strata (*p* for interaction >0.05). Lastly, in the substitution analyses, replacing one serving per day of processed meat by one serving per day of red meat, poultry, or other important protein sources (fish, legumes, dairy, and nuts) was associated with a lower risk of agility. The results were in the same direction for impaired lower-extremity function, although they did not achieve statistical significance in all cases (Table 4).

**Table 4:** HR and 95% CI for replacing one serving per day of processed meat for another source of protein

Substitution	Impairment in agility	Impairment in lower-extremity function
Red meat for processed meat	0.96 (0.86–1.07)	0.63 (0.56–0.72)
Poultry for processed meat	0.70 (0.64–0.77)	1.03 (0.95–1.10)
Fish for processed meat	0.82 (0.75–0.90)	0.68 (0.63–0.73)
Legumes for processed meat	0.86 (0.76–0.99)	0.97 (0.79–1.20)
Dairy for processed meat	0.75 (0.65–0.88)	0.85 (0.68–1.06)
Nuts for processed meat	0.59 (0.57–0.61)	0.60 (0.55–0.65)

Model is adjusted for age, sex, educational level (≤primary, secondary, or university), smoking status (never smoked, former smoker, or current smoker), alcohol intake (quintiles of g/day), energy intake (quintiles of kcal/day), BMI (<25, 25 < 30, ≥30 kg/m^2^), sedentary behavior (quintiles of hours/week watching television), and morbidity (cognitive impairment, osteomuscular disease, cardiovascular disease, cancer, chronic lung disease, or depression)

One serving of red meat, processed meat, poultry, and fish = 100 g; one serving of legumes = 75 g; one serving of dairy = 150 g; one serving of nuts = 30 g

*BMI* body mass index, *CI* confidence interval, *HR* hazard ratio

## Discussion

In this study, a higher habitual consumption of processed meat was associated with increased risk of impaired agility and lower-extremity function. In addition, substitution of processed meat with fish, legumes, dairy, or nuts was associated with reduced risk of functional impairment. We tried to capture the broad dimension of physical functioning using several self-reported and performance-based measurements, since self-reported measures assess the perception of the ability to perform a functional task and performance-based measures assess the ability to complete a task.

No significant associations between red meat or poultry and physical function were found. Since red meat has been strongly associated with increased mortality [17], it is possible that the low consumption in this cohort was not enough to reveal a detrimental impact on physical functioning. Similarly, it is possible that the intake of poultry was not high enough to show a plausible beneficial effect; however, substitution analyses showed that replacement of processed meat for poultry reduced the risk of impaired function.

Few studies have investigated the association between meat intake and physical functioning. A recent paper among participants of the Framingham Offspring study investigated the association of the consumption of several protein sources with skeletal muscle mass and functional decline [22]. A higher intake of red meat and poultry was associated with higher muscle mass, especially among women. However, in line with our results, consumption of red meat and poultry was not significantly associated with developing two or more functional impairments; only when this consumption was combined with high physical activity were there beneficial effects on functional status [22]. In addition, in a randomized trial, participants receiving a protein-enriched diet through the addition of 160 g/day of lean red meat combined with resistance training showed a greater increase in leg-extension muscle strength after 4 months, compared with the control group that only underwent resistance training [32]. However, muscle function, measured through a four-square step test, a times-up-and-go test, and a 30-s sit-to-stand test, did not differ between the two randomized groups. We stratified our analyses by physical activity to compare our results with these findings, but we did not observe any joint association between meat intake and high physical activity level on subsequent physical function. In addition, the investigators of the Framingham Third Generation Study identified clusters of people who consumed a high amount of red meat and chicken; these clusters did not show an association with muscle mass or muscle strength [33].

There are several potential mechanisms for the association of processed meat and functional impairment. Protein is an important component of meat; however, meat and especially processed meat also contain a considerable amount of saturated and *trans* fat. These types of fat have previously been shown to increase inflammation [34], which may subsequently reduce physical functioning [35]. Additionally, compared to red meat and poultry, the content of sodium and nitrites is much higher in processed meat [36, 37]. Sodium and nitrites may increase cardiovascular disease risk through increased blood pressure and endothelial dysfunction [38, 39]. This suggests that the beneficial effect of the high-quality protein in meat might be counterbalanced by the high content of saturated and *trans* fat, sodium, and nitrite in processed meat. This hypothesis is supported because the risk of physical function impairment lowered when processed meat was replaced by other protein sources.

Fish is a common substitute for meat. Processed meat was inversely correlated with fish consumption. Since in our cohort fish consumption was relatively high (67.3 g/day) and this food is an important source of omega-3 fatty acids with anti-inflammatory effects [34], it is plausible that some of the detrimental effect of processed meat was due to the lack of consumption of fish. However, when we adjusted the analyses for this and other food groups, the associations still held.

The strengths of this study are its prospective design, the estimation of habitual meat intake through a validated diet history, and the repeated measurements during follow-up, which allowed us to calculate the cumulative average consumption and to update confounding factors over time, which reduces the number of random errors and improves the precision of estimates. However, certain misreporting and misclassification of dietary intake cannot be ruled out, even though we excluded participants with an implausibly high or low energy intake level. Some further limitations should also be acknowledged. The self-reported physical function measures may be less reliable than objective measurements. However, reported function has been shown to predict early decline in performance and early disease [40]. Finally, as in any observational study, some residual confounding may persist.

## Conclusions

A higher consumption of processed meat, but not red meat or poultry, was associated with increased risk of impaired agility and lower-extremity function in older adults. We did not find evidence that meat, despite its high content of protein, has a protective effect on impairment in physical functioning. These results should be confirmed in future research in countries with a higher meat intake.

## Additional file

Additional file 1: **Table S1.** HR and 95% CI for the association between meat consumption and impairment in mobility. (PDF 173 kb)

## Abbreviations

BMI: Body mass index
CI: Confidence interval
HR: Hazard ratio
MMSE: Mini-Mental State Examination
SPPB: Short Physical Performance Battery
