# Supplementary material for: Consumption of meat in relation to physical functioning in the Seniors-ENRICA cohort
Source: BMC Med. 2018 Apr 5;16:50. doi: 10.1186/s12916-018-1036-4 (PMC5887175; doi:10.1186/s12916-018-1036-4)
Supplement: Supplementary file 1 — Table S1. HR and 95% CI for the association between meat consumption and impairment in mobility. (PDF 173 kb) [file 12916_2018_1036_MOESM1_ESM.pdf]

ADDITIONAL TABLE

Additional table 1. HR and 95% CI for the association between meat consumption and impairment in mobility.

	Meat intake			P-trend	Continuous
	Tertile 1	Tertile 2	Tertile 3		per 100 g/d
N participants	893	894	894		2681
**Processed meat**					
Mean intake, g/d	8.5 ± 5.9	27.9 ± 6.0	69.6 ± 38.0		35.3 ± 34.0
Impairment in agility, n/person-years	148/4,429	165/4,652	142/4,661		455/13,742
Model 1	Reference	1.09 (0.88; 1.37)	1.11 (0.88; 1.41)	0.36	1.01 (0.76; 1.35)
Model 2	Reference	1.11 (0.88; 1.38)	1.03 (0.81; 1.31)	0.80	0.92 (0.68; 1.25)
Model 3	Reference	1.12 (0.89; 1.41)	1.04 (0.81; 1.33)	0.76	0.92 (0.67; 1.25)
**Red meat**					
Mean intake, g/d	7.5 ± 5.4	26.3 ± 5.9	63.8 ± 27.4		32.5 ± 28.6
Impairment in agility, n/person-years	165/4,536	173/4,614	117/4,593		455/13,742
Model 1	Reference	1.19 (0.96; 1.48)	1.01 (0.79; 1.29)	0.81	0.93 (0.63; 1.38)
Model 2	Reference	1.28 (1.02; 1.59)	1.11 (0.86; 1.43)	0.31	1.01 (0.68; 1.50)
Model 3	Reference	1.25 (1.00; 1.57)	1.10 (0.85; 1.42)	0.39	1.00 (0.67; 1.49)
**Poultry**					
Mean intake, g/d	9.0 ± 6.6	28.5 ± 5.4	64.2 ± 28.6		33.9 ± 28.6
Impairment in agility, n/person-years	144/4,485	159/4,655	152/4,601		455/13,742
Model 1	Reference	1.11 (0.89; 1.40)	1.12 (0.89; 1.40)	0.35	1.15 (0.82; 1.61)
Model 2	Reference	1.10 (0.88; 1.38)	1.09 (0.87; 1.38)	0.45	1.06 (0.76; 1.50)
Model 3	Reference	1.13 (0.90; 1.43)	1.11 (0.87; 1.40)	0.40	1.08 (0.76; 1.53)

Model 1: adjusted for age and sex
Model 2: adjusted for age, sex, educational level (≤primary, secondary, university), smoking status (never smoker, former smoker, current smoker), alcohol intake (quintiles of g/d), energy intake (quintiles of kcal/d), BMI (<25, 25-<30, ≥30 kg/m^2), sedentary behaviour (quintiles of h/wk watching TV) and morbidity (cognitive impairment, osteomuscular disease, cardiovascular disease, cancer, chronic lung disease, and depression).
Model 3: additionally adjusted for vegetables, legumes, fruits, nuts, cereals, dairy, and fish consumption (quintiles of g/d).
